# Validation of the French ADNM-20 in the assessment of emotional difficulties resulting from COVID-19 quarantine and outbreak

**DOI:** 10.1186/s40359-021-00683-7

**Published:** 2021-11-13

**Authors:** A. Vancappel, E. Jansen, R. Bachem, A. Bray, L. Egreteau, C. Réveillère, A. Maercker, W. El-Hage

**Affiliations:** 1 Pôle de Psychiatrie-Addictologie, Clinique Psychiatrique Universitaire, CHRU de Tours, Tours, France; 2 Département de Psychologie, EE 1901 Qualipsy, Qualité de vie et Santé Psychologique, Université de Tours, Tours, France; 3 UMR 1253, iBrain, Inserm, Université de Tours, Tours, France; 4 Universität Zürich, Zurich, Switzerland

**Keywords:** Adjustment disorder, Outbreak, COVID-19, Quarantine

## Abstract

**Background:**

Multiple psychological consequences of the COVID-19 outbreak and quarantine have been described. However, there is a lack of global conceptualization. We argue that the stressful aspects of the situation, the multiple environmental consequences of the outbreak, and the diversity of symptoms observed in such a situation, suggest that Adjustment disorder (AD) is a promising way to conceptualize the psychological consequences of the outbreak and quarantine. The first aim of the study was to validate the French version of the ADNM. The second aim was to set out adjustment difficulties resulting from COVID-19 outbreak and quarantine.

**Method:**

We recruited 1010 (840 women, 170 men) who consented online to participate. They filled out the French ADNM, visual analogic scales, HADS, IES, and the COPE, to evaluate coping strategies.

**Results:**

We confirmed the factor structure of the ADNM and we found good psychometric properties. We found that 61.3% of participants presented an adjustment disorder related to COVID-19 outbreak. We found multiple risk factors and protective factors to AD due to quarantine and outbreak. We also identified the coping strategies negatively and positively associated with AD.

**Conclusion:**

Adjustment disorder is a relevant concept to understand psychological manifestations caused by quarantine and outbreak. The French ANDM has good psychometric properties to evaluate such manifestations. The association between coping strategies and AD symptoms suggest that CBT may be the best intervention to help people suffering from AD.

**Supplementary Information:**

The online version contains supplementary material available at 10.1186/s40359-021-00683-7.

## Introduction

The 21st January 2020, the World Health Organization published its first report relating the existence of coronavirus disease 2019 (COVID-19). On May the 16th 2020, the virus had spread in 216 different countries and 4,396,392 cases have been confirmed [1]. France was strongly impacted with large and growing numbers of confirmed cases (141,919) and deaths (27,529) [2]. To slow down the virus spread, the French government imposed quarantine measures for two months, from March 17th to May 11th.

A recent meta-analysis including the studies performed during the past disease outbreaks, underlined that quarantine during an outbreak is associated with the development of new symptoms of anxiety, depression, and post-traumatic stress [3]. Different studies explored the impact of the outbreak and quarantine on the general population for the COVID-19 outbreak. Studies carried out in China reported depressive and post-traumatic symptoms among students [4]. They also set out 35% of moderate to severe stress [5], anxiety, depression and post-traumatic symptoms [6] among the general population. In Spain, a study reported 18.7% of depressive manifestations, 21.6% of anxiety symptoms, and 15.8% of PTSD symptoms in a sample of 3480 participants [7]. Another study also reported a more important level of anxiety and depressive symptoms after a few days of stay at home order [8]. An Italian survey highlighted 17% of high and 15.8% of very high depression scores, 7.2% of high and 11.5% of very high anxiety scores, and 14.6% of high and 16.6% of very high stress scores through Depression, Anxiety and Stress Scale (DASS) [9]. Recently a meta-analysis including 13 studies set out anxiety, anger, stress, post-traumatic, and loneliness symptoms [10]. To the best of our knowledge, for the moment, no studies have been published among the French population.

Up to now, studies related to COVID-19 focused essentially on the description of the psychological consequences of outbreak and quarantine, and the identification of risk factors. Multiple risk factors have been identified: being a woman [5, 6, 9], having physical symptoms [6, 7], a history medical problems [7, 9], being a student, perceived low health, a lack of actual information [6], being younger (18–30) and older (+60), having a family member working away from home [5], living in a more impacted area [4] and having sick relatives [7]. However, no work has proposed a general conceptualization of the manifestations. Especially, to our knowledge, no study has considered the multiplicity of symptoms observed during the outbreak. We propose that the best way to understand the psychological consequences of outbreak and quarantine, is to conceptualize these manifestations as an adjustment disorder (AD). According to the ICD-11, AD is composed of core symptoms: preoccupations and failure adapt, with potentially additional symptoms (e.g., anxiety) [11]. AD is also defined as an emotional and behavioral response to identified stress factors. Multiple subtypes have been proposed and can include one or multiple manifestations such as depressive mood, anxiety, or impulse disturbance [12]. We argue that AD fits well for manifestations observed during the outbreak and quarantine. Firstly, the outbreak and quarantine constitute a stressful event. Secondly, the outbreak can create secondary stressful events, which can lead as well to emotional difficulties. For instance, more than 10 million French people have been partially unemployed during a few weeks [13]. Studies have already set out that financial difficulties is a determining risk factor for emotional difficulties [3]. Quarantine also leads to isolation, end of a leisure activities (such as team sport), and even divorce [14]. Finally, the multiplicity of the observed symptoms also fits well the diversity of manifestations observed in AD.

Adjustment disorder has been studied poorly for the moment, while it is the 7th most used diagnosis [15]. To enable clinicians to assess AD and researchers to improve conceptualization, a scale has been developed: Adjustment disorder new module (ADNM) [16]. To our knowledge, the ADNM is the only scale that evaluates AD. The last version [17] contains 20 items, 19 items evaluating the symptoms and the last one evaluating functional impairment. The scale measures six symptom areas: preoccupation with the stressor, failure to adapt, avoidance, depression, anxiety, and impulsivity. Multiple studies have focused on the factor structure of adjustment disorder. Some experiments have shown a good fit of a one-factor model [17, 18], a 6-factor structure [19], a bi-factorial-structure [1 general factor and 6 specific factors] [20] and bi-factorial model of main symptoms (preoccupations and failure to adapt) [18, 19]. Thus, the one-dimensionality of AD may help explain the global impact of the outbreak and quarantine. Its multidimensionality could explain the diversity of the observed manifestations.

Thus, the primary aim of the study was to validate the psychometric properties of the French version of the ADNM. The secondary aims of the protocol were (a) to demonstrate the relevance of AD as a conceptualization of psychological consequences due to outbreak and quarantine and (b) to evaluate the risk factors and the coping strategies associated with AD in the context of COVID-19 pandemic.

## Method

### Participants

Participants were recruited through social networks. Participation required reading an information-note online, checking a box to consent to participate, and choosing to either continue with the study or decline to proceed. The experiment and informed consent procedures were approved by the ethics committee of the University (Comité d’Ethique de la Recherche Tours-Poitiers, n°2020-04-02). All methods were performed in accordance with the relevant guidelines and regulations imposed by this institution.

### Procedure

To perform the study, we used the *Adjustment Disorder New Module* (ADNM) [17] that was translated in French. We used translation/back-translation method to develop the French version of ADNM [21]. First, two translations were performed by two authors (AV and WEH) who combined their work to develop the first version of the ADNM. A third author (EJ) back translated it into English literally to convey the meaning of the translation. A fourth author (RB) compared the back-translation with the original text and highlighted some issues needing to be clarified. An extensive discussion took place among all the bilingual authors who agreed on minor changes, reconciling any meaningful differences between the two. The authors modified accordingly the questionnaire to include these changes, resulting in the final French version of the ADNM scale for validation testing (Additional file 1: ADNM 20 items – Trouble de l’Adaptation Nouveau Module 20). "Following consent, participants responded to sociodemographic questions. Then, they filled out successively a series of online questionnaires. Data were collected between April the 2nd and May the 10th, 2020. Participants had to be over 18 years of age to perform the study. Data were collected with Sphinx Software, 4th version. Only fully completed responses were gathered by the software.

### Measures

#### Adjustment disorder new module (ADNM)

The ADNM is composed of two parts. In the first part, participants have to indicate the stressful events that occurred during the past 2 years and which burden(ed) them during the last 6 months. Due to the context, “quarantine due to outbreak” was added to the initially proposed events. Then participants had to indicate the most burdensome event(s). For the rest of the study, we will refer to these events as main events. Then, they had to answer multiple questions related to these events with a four points-Likert scale from 1 (never) to 4 (often). For each question, they also had to indicate the duration: less than 1 month, 1–6 months, 6 months to 2 years. A previous study has set out good psychometric properties among burglary victims (α = 0.94) [22].

Participants who reported “quarantine due to outbreak” as one of the main events answered 16 complementary questions: 15 analogic visual questions plus 1 dichotomous question (see Additional file 2: Visual analogic scales). These questions were related to their general behavior during quarantine. Participants had to answer from 0 (not at all) to 10 (perfectly).

#### Hospital Anxiety Depression scale (HAD)

The HAD is a self-report questionnaire that evaluates depression and anxiety [23]. Seven questions are related to anxiety and seven related to depression. Participants had to answer on a 4 points Likert scale. One item for example is, “I took as pleasure as I used to”. The French version showed good psychometric properties (Cronbach alpha from 0.67 to 0.90) [24].

#### Impact Event Scale 6 items (IES-6)

The IES-6 is a self-questionnaire. Participants had to answer questions related to the main events they identified on a 5 points Likert scale [25]. Experimental studies demonstrated good psychometric properties (Cronbach α = 0.80).

#### Brief COPE

This self-questionnaire evaluates coping strategies [26]. It is composed of 28 items. It assesses active coping, planning, instrumental support, emotional support, emotional expression, positive reappraisal, acceptance, denial, blame, humor, religion, distraction, substance use, behavioral disengagement. Each dimension is evaluated with two questions. The scale demonstrates good psychometric properties in the French population [26].

### Data analysis

First, we performed multiple analysis to assess the psychometric proprieties of the ADNM. We used confirmatory factor analysis to assess different models presented in the literature. Five models evaluated the 19 symptoms items of the ADNM. The first model was a 6 factors model proposed by Einsle [16]. The second model contains 6 factors plus 1 general factor model, proposed by Lorenz [20]. The third model was a one-factor model proposed by Glaesmer [17]. Model 4 was a 5-factor model proposed by Lorenz [20]. In this model, depression and anxiety are combined as one affective factor. The next model was a 6 first-order factors and one global second-order factor. This model was proposed by Lorenz et al. (2017). A 2-factor model [19] was also assessed on the 7 items related to the core symptoms (preoccupations and failure to adapt). Then, we kept the best model and we added correlated errors between items 9 and 12 as suggested by Lorenz [18]. We used Chi^2^, comparative fit index (CFI), Tucker-Lewis (TLI), and root-mean-square error of approximation (RMSEA). RMSEA under 0.08 and CFI and TLI above 0.90 suggest a god fit model [27]. Strong fit can be considered with CFI and TLI greater than 0.95 and RMSEA lower than 0.06 [28].

Following the last guidelines [29], we used Mc Donald’s Omega to asses internal consistency. Because the anxiety subscale contains only two items, we performed Cronbach’s alpha instead of Mc Donald’s Omega. We also computed correlational analysis (Bravais-Pearson) to assess the concurrent validity of ADNM.

Then, we explored descriptive analysis to describe the emotional influence of the different events. We also computed correlational analysis (Bravais-Pearson) to assess risk factors in Adjustment disorder, and during the quarantine. We distinguished participants who reported quarantine as a main event from those who did not report it as a main event. We used T-test student and Cohen’s d to evaluate the sex differences and the influence of isolation during the quarantine. We also used T-tests and Cohen’s d to compare participants who reported quarantine as one of the most burden events and those who did not. All analyses were performed with IBM: SPSS/AMOS 23th version.

## Results

### Factor analysis

**Fig. 1:**
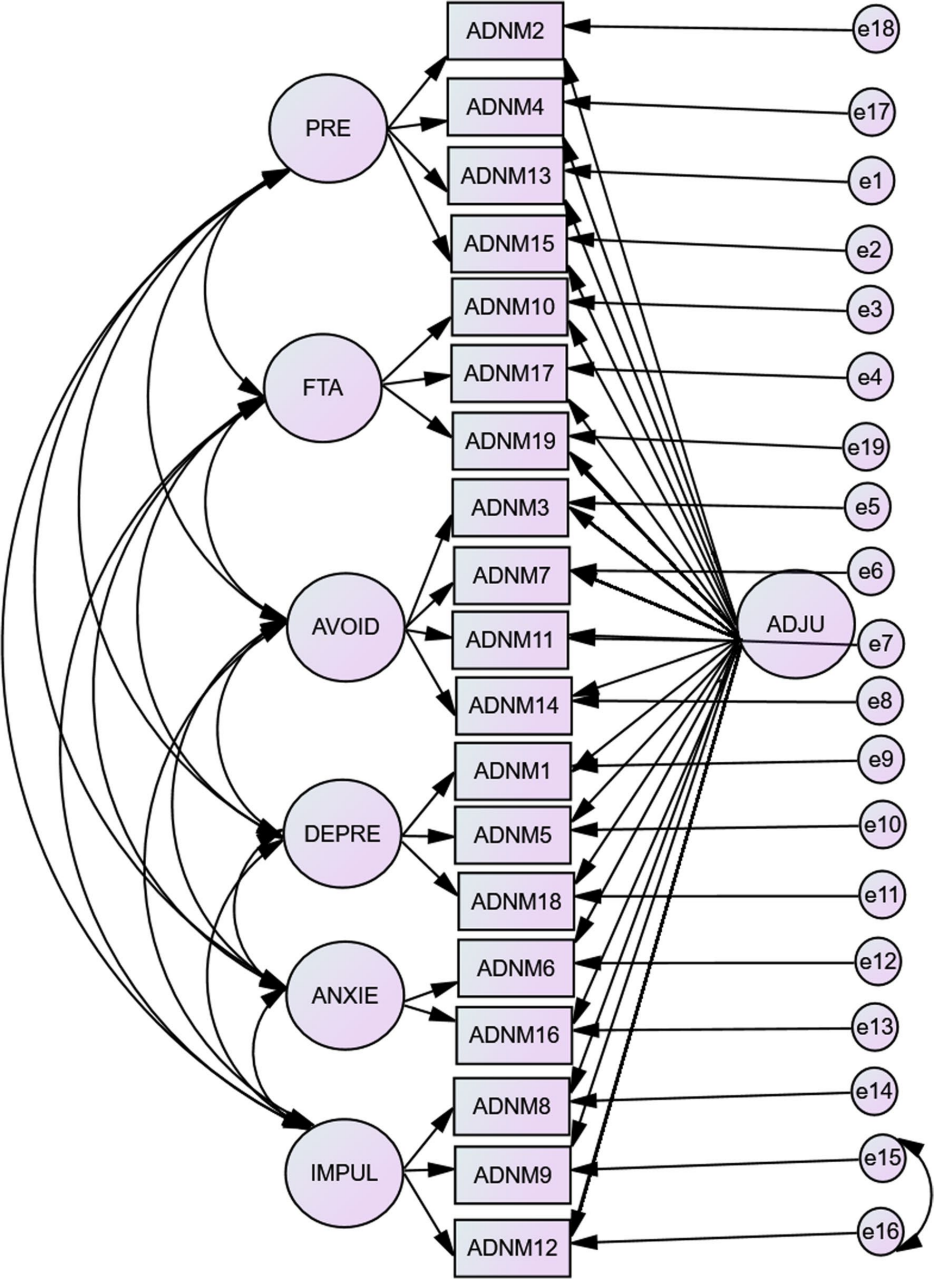
Schematic representation of the 7th model. *ADJU* adjustment factor, *PRE* preoccupations, *FTA* failure to adapt, *AVOID* avoidance, *DEPRE* depressive mood, *ANXIE* anxiety, *IMPUL* impulse disturbance

The results of the confirmatory factor analysis are displayed in Table 1. All the Chi^2^ were significant, but this cannot lead to the rejection of the models, as the sample is large (Tanaka, 1987). The models 3 (1-factor model), 5 (2 core factors for 7 main symptoms), and 6 (6 first-order factors and 1 s-order factor) were weak with TLI and CFI under 0.90 and RMSEA above 0.08. Model 1 (6 factors model), model 2 (6 factors + 1 general factor model), and model 4 (5-factor model) were acceptable with CFI and TLI around 0.90 and RMSEA under 0.08. The best one was the second model. Thus, we added in the 7th model, a correlation between items 9 and 12 errors due to the closeness of the items proposed by Lorenz [18]. This increased the fit of the model (CFI = 0.966, TLI = 0.950, RMSEA = 0.049). According to the different indices, this makes model 7 a great model fitting the data (see Fig. 1).

**Table 1:** Fit indices for structure adjustment disorder (N = 1010)

Model	Structure model	Chi^2^	Df	CFI	TLI	RMSEA
Model 1	6 factors	861.6*	137	.912	.890	.072
Model 2	6 factors + 1 general factor	439.7*	118	.961	.943	.520
Model 3	1 general factor	1541.1*	152	.831	.810	.095
Model 4	5 factors, anxiety depression combined	908.2*	142	.901	.888	.073
Model 5	2 factors on the 7 main symptoms	862.7*	14	.682	.523	.245
Model 6	6 first order factors and 1 s order factor	1791.3*	148	.800	.769	.105
Model 7	Model 2 + correlation between item 9 and 12’s error	397.1	117	.966	.950	.049

*CFI* Comparative Fit Index, *TLI* Tucker-Lewis Index, *RMSEA* Root-Mean-Square Error of Approximation

*p < .001

### Internal consistency

We evaluated the internal consistency using Mc Donald Omega and Cronbach alpha depending on the statistical possibilities. We found a good internal consistency for the entire scale (*ω* = 0.918). However, we found weaker internal consistencies for the different subscales: preoccupations (*ω* = 0.852), failure to adapt (*ω* = 0.669), avoidance (*ω* = 0.727), depressive mood (*ω* = 0.591), impulse disturbance (*ω* = 0.810) and anxiety (*α* = 0.613).

### The concurrent validity of ADNM

We first evaluated the correlations, on the complete sample, between ANDM, HAD, and IES-6 scores. We found strong correlations between ADNM and anxiety-HAD scores (*r* = 0.744, *p* < 0.001), depression-HAD scores (*r* = 0.646, *p* < 0.001) and IES-6 scores (*r* = 0.723; *p* < 0.001). This suggests a good concurrent validity of the ADNM within the French population.

### Descriptive analysis

Among the 1010 participants (840 women, 170 men), the mean age was 34.79 (SD = 13.6). The repartition of the burdensome events is presented in Table 2. A majority (78.6%) of the participants reported the quarantine as a burden. However, only 375 participants reported quarantine as one of the most burdensome events. To answer the multiple questions of the study, we cut the sample by distinguishing the participants who reported quarantine as one of the most burdensome events (QB group; 318 women, 57 men, age 35.26 ± 13.94) from the participants who did not report quarantine as one of the most burdensome events (nQB group;522 women, 113 men, age 34.5 ± 13.40).

**Table 2:** Repartition of the burden events

Event	Percentage	Number of participants
Divorce separation	19.9	201
Familial conflict	27.7	280
Conflict in work life	17.5	177
Conflict with the neighbors	6.1	62
Illness of a loved one	31.2	315
Death of a loved one	28.3	286
Adjustment due to retirement	1.3	13
Unemployment	8.3	84
Too much/too little work	40.9	413
Pressure to meet deadlines/time pressure	35.6	360
Moving to a new home	26.9	372
Financial problems	19.2	194
Own serious illness	8.6	87
Serious accident	3.5	35
Assault	8	81
Termination of an important leisure activity	17.6	178
Quarantine due to outbreak	78.6	794
Any other stressful event	25.5	258

The descriptive data of the QB and nQB groups are presented in Table 3. In general, the QB group and nQB group have similar scores. This suggests that adjustment disorder is a relevant conceptualization of the impact of quarantine. When applying the cut-off scores proposed of 47.5 at the ADNM [22], we found that 54% (343 participants) of the nQB group and 61.3% (230 participants) of the QB presented an AD.

**Table 3:** Descriptive analyses of participants who reported quarantine as the most burdensome events (QB group) and participants who did not report quarantine as one of the most burdensome events (nQB group)

	Group QB		Group nQB	
	Range	Mean ± SD	Range	Mean ± SD
N 1M	0–20	13.32 ± 7.36	0–20	9.79 ± 7.11
N 1–6M	0–20	4.10 ± 5.87	0–20	4.60 ± 5.72
N 6–24M	0–20	2.58 ± 5.02	0–20	5.61 ± 6.76
**ADNM**				
Preoccupations	4–16	10.56 ± -3.51	4–16	10.50 ± 3.52
Failure to adapt	3–12	7.24 ± 2.55	3–12	6.84 ± 2.59
Avoidance	4–16	9.81 ± 3.40	4–16	10.06 ± 3.43
Depressive mood	3–12	7.70 ± 2.27	3–12	7.32 ± 2.31
Anxiety	2–8	5.18 ± 1.84	2–8	5.01 ± 1.96
Impulse disturbance	3–12	7.47 ± 2.71	3–12	7.32 ± 2.77
Item 20	1–4	2.63 ± 1.11	1–4	2.38 ± 1.16
Total	20–80	50.64 ± 13.63	20–80	49.43 ± 13.81
HAD Anxiety	0–21	9.12 ± 4.42	0–21	8.83 ± 4.36
HAD Depression	0–20	6.58 ± 4.22	0–21	5.78 ± 4.32
**IES-6**				
Intrusion	0–8	4.32 ± 2.42	0–8	4.17 ± 2.51
Avoidance	0–8	3.34 ± 2.31	0–8	3.35 ± 2.36
Hyperarousal	0–8	3.37 ± 2.43	0–8	2.92 ± 2.45
Total	0–24	11.03 ± 6.05	0–24	10.45 ± 6.26
**Brief COPE**				
Active coping	2–8	4.69 ± 1.51	2–8	4.94 ± 1.64
Planning	2–8	4.85 ± 1.72	2–8	5.03 ± 1.82
Instrumental support	2–8	4.55 ± 1.71	2–8	4.65 ± 1.85
Emotional support	2–8	4.89 ± 1.73	2–8	4.79 ± 1.75
Emotional expression	2–8	4.69 ± 1.57	2–8	4.69 ± 1.61
Positive reappraisal	2–8	4.97 ± 1.74	2–8	5.02 ± 1.83
Acceptation	2–8	5.68 ± 1.69	2–8	5.74 ± 1.72
Denial	2–8	3.17 ± 1.57	2–8	3.04 ± 1.55
Blame	2–8	4.14 ± 1.75	2–8	4.52 ± 1.74
Humor	2–8	3.38 ± 1.48	2–8	3.14 ± 1.39
Religion	2–8	3.08 ± 1.67	2–8	3.20 ± 1.72
Distraction	2–8	5.26 ± 1.62	2–8	5.18 ± 1.61
Substance use	2–8	2.76 ± 1.32	2–8	2.83 ± 1.47
Behavioral disengagement	2–8	3.40 ± 1.47	2–8	3.32 ± 1.50
**Quarantine**				
Activity	0–10	6.92 ± 2.46	–	–
Sleep	0–10	5.06 ± 2.93	–	–
Alimentation	0–10	6.36 ± 2.51	–	–
Positivity	0–10	6.15 ± 2.56	–	–
Relax	0–10	5.49 ± 2.63	–	–
Stress management	0–10	5.70 ± 2.43	–	–
Information	0–10	6.56 ± 2.30	–	–
Screen	0–10	4.05 ± 2.89	–	–
Physical activity	0–10	4.85 ± 3.25	–	–
Social contact	0–10	7.07 ± 2.15	–	–
Solidary activity	0–10	2.77 ± 3.08	–	–
Substance use	0–10	1.95 ± 2.84	–	–
Professional activity	0–10	5.91 ± 3.92	–	–
Respect	0–10	9.22 ± 1.57	–	–

*ADNM* Adjustment Disorder New Module, *IES-6* Impact Event Scale 6 items, *HAD* Hospital Anxiety Depression Scale, *N 1M* number ADNM symptoms present for less than 1 month, *N 1–6M* number of ADNM symptoms present between 1 and 6 months, *N 6–24 M* number of ADNM symptoms present between 6 and 24 months

### Correlation analysis

#### Risk factors for adjustment disorder

We also performed Pearson correlation analysis to evaluate the association between ADNM scores and age and coping strategies evaluated by the Brief COPE. First, age was not significantly associated with ADNM scores (*r* = − 0.19, *p* = 0.539). The same result was observed in the QB group (*r* = − 0.41; *p* = 0.432). For the entire sample, ADNM scores were positively associated with instrumental support (r = 0.144; *p* < 0.001), emotional support (*r* = 0.277; *p* < 0.001), denial (*r* = 0.378; *p* < 0.001), blame (*r* = 0.444; *p* < 0.001), religion (*r* = 0.0104; *p* < 0.001), substance use (*r* = 0.217; *p* < 0.001) and behavioral disengagement (*r* = 0.396; *p* < 0.001). The more participants used these coping strategies, the more they had adjustment difficulties. ADNM score was negatively correlated to planning (*r* = − 0.111; *p* < 0.001), positive reframing (*r* = − 0.373; *p* < 0.001), acceptance (*r* = − 0.448; *p* < 0.001), and humor (*r* = − 0.327; *p* < 0.001). This suggest that the more participants used these strategies, the less they had adjustment difficulties. Finally, no correlation was identified between ADNM scores and expression feeling (*r* = − 0.040; *p* = 0.205) and distraction (*r* = 0.043; *p* = 0.76).

#### Risk factors for adjustment disorder due to quarantine

We performed correlation analysis between ADNM score and visual analogic scores. This analysis was only performed on the QB group as only those participants undertook the visual analogic scales. We found a negative association between ADNM scores and boredom (*r* = − 0.298; *p* < 0.001), sleep rhythm (*r* = − 0.394; *p* < 0.001), stable alimentation (*r* = − 0.341; *p* < 0.001), ability to relax (*r* = − 0.492; *p* < 0.001), preoccupation management (*r* = 0.570; *p* < 0.001), communication abilities (*r* = − 0.341; *p* < 0.001), clear information get (*r* = − 0.195; *p* < 0.001), management of screen time (*r* = − 0.244, *p* < 0.001), regular physical activity (*r* = − 0.225; *p* < 0.001), social contact (*r* = − 0.344; *p* < 0.001), participations in united activities (*r* = − 0.143;p < 0.001) and professional activity (*r* = − 0.224; *p* < 0.001). We did not find association between ADNM and substance increase (*r* = 0.050; *p* = 0.332) and quarantine violation (*r* = − 0.008; p = 0.882).

### Group comparisons

Then, we performed group comparisons. We first compared men and women. On the whole sample, we found that women have higher mean total score than men on ADNM (*T* = 4.52; *p* < 0.001; d = 0.37). When we computed this analysis on the QB group, we did not find a significant effect (*T* = 1.313; *p* = 0.190). We also compared within the QB group, the ADNM scores between participants who were alone during quarantine and those who were not alone. We did not find a significant difference between the two groups (*T* = -1.504; *p* = 0.134).

We performed multiple comparisons t-tests between the QB group and the nQB group. We used Bonferroni correction to adjust the p-value. We performed 11 comparisons, thus the new p-value was 0.0045. We compared age, the six sub-dimensions of the ADNM, the score at 20th item of the ADNM (assessing functional impairment), HAD depression, HAD anxiety, and IES-6 score. Only HAD depression scores (*T* = 2.882; *p* < 0.005; d = 0.19) and the 20th item of the ADNM (*T* = 3.346; *p* < 0.001; d = 0.22) scores were significantly different. Participants of the QB group presented higher scores than participants in the nQB group. This suggests that quarantine compared to other events leads to more functional impairments and more depressive affects. It also suggests that quarantine leads to comparable difficulties as observed in other stressful events.

## Discussion

This study was conducted in the context of COVID-19 pandemic. Firstly, we wanted to evaluate the psychometric properties of the French ADNM. Confirmatory factor analysis suggests that the bi-factorial model including a general factor and 6 complementary factors best fits the data as found by Lorenz [20]. This tends to confirm the one-dimensionality and multidimensionality of AD. However, the bi-factorial performed on the main symptoms did not fit well as opposed to the previous study that reported a good fit [18–20]. We found a good internal consistency for the entire ADNM. However, contrasting with the results of the initial version [17], we did not find a great internal consistency for some subscales: failure to adapt, depressive mood and anxiety. We found good concurrent validity with strong correlations between ADNM scores and anxiety, depression, and post-traumatic levels.

Secondly, we wanted to evaluate the relevance of conceptualizing psychological consequences of quarantine and outbreak as AD. We found that participants presenting difficulties related to the COVID-19 outbreak, scored the same on the sub-dimensions of ADNM. However, they performed higher on the 20th item and the HAD depression scores. This means that outbreak and quarantine lead to significant adjustment disorder symptoms. This also suggests that this event is slightly more impacting than others are. This tends to be confirmed by a higher prevalence of AD in the QB group.

Moreover, we evaluated risk factors for AD in general, and among participants impacted by COVID-19 quarantine and outbreak. We found that women scored higher at the ADNM. This was only significant for the entire sample but not for the QB group. This is in disagreement with the results presented in other countries where women tend to be more emotionally impacted by COVID-19 sanitary crisis than men [5, 6, 9]. Contrary to another study we also did not find that age or being alone during the quarantine were risk factors [6]. However, as reported by previous studies, we found that boredom and lack of clear information were significant risk factors [3]. We also found multiple protector factors, such as sleep rhythm, stable alimentation, ability to relax, ability to manage worries, communication abilities, screen time management, regular physical activity, social contact, participation in united activities, and keeping a professional activity. This offers multiple axes of intervention to help people adjust to quarantine and outbreak. Working from home may be encouraged. Psychoeducation on alimentation, sleep functioning, physical activity and screen effects may be proposed. Communication training and relaxation techniques may also be relevant. Clear information, distant social contact, and united activities may be promoted.

Finally, results highlighted multiple associations between AD and coping strategies. On one side, results suggest that the more people use instrumental support, emotional support, denial, blame, religion, substance use, and behavioral disengagement, the more they have adjustment difficulties. On the other side, the more they use planning, positive reframing, acceptance, and humor, the less they have adjustment difficulties. This is partly congruent with the results of Einsle [16] who found that emotion-oriented and somewhat proactive active coping are positively associated with AD.

These results have multiple implications for further psychotherapeutic trials. First, the association between AD and blame, humor, and cognitive reframing suggest the relevance of cognitive therapy for treating AD [30]. Second, the association with denial, behavioral disengagement, and acceptance may suggest the relevance of exposure therapy. Indeed, a part of exposure therapy is to develop the emotional digestion of the event [31]. This is also congruent with a previous work that suggests an adaptation of classical models of post-traumatic stress disorder to AD [32]. The association with planning may also suggest the relevance of problem-solving techniques [30]. As the psychological consequences of quarantine and outbreak fit well the concept of AD, these interventions may also be relevant for patients presenting such difficulties. On the contrary, the association between AD and emotional support may question the efficacy and even the danger of psychological support intervention. Altogether, this information suggests that Cognitive Behavioral Therapy (CBT) might be the best intervention to help people suffering from AD. This is congruent with the recommendation of psychotherapy instead of drug prescriptions even if randomized protocols for AD are missing [33]. However, controlled randomized trials should be perform among patients suffering from AD to highlight the relevance of such intervention for this disorder.

Our study presents some strengths. First, we have a large sample that permits an important concurrent validity. Second, we confirmed the structure of the ADNM with participants presenting multiple life events. Finally, we used highly used and well-validated scales to assess the association between ADNM and depression, anxiety and post-traumatic symptoms.

The study has few limits. First, participants are mostly women. Thus, it would be interesting to perform another study, respecting the balance between men and women. Then, the evaluation of emotional difficulties is performed with self-questionnaire. Diagnostic interview would be more in-depth evaluations. It would allow more rigorous assessment of the presence of AD. Moreover, the study was performed online and this could constitute a bias. Finally, the test–retest stability has not been evaluated in this study. Such evaluation would be required to complete the psychometric properties of ADNM.

## Conclusion

Adjustment disorder is a relevant concept to understand psychological manifestations caused by quarantine and outbreaks. The French ANDM has good psychometric properties to evaluate such manifestations. Psychotherapeutic interventions via telemedicine need to be promoted in the pandemic context to help people suffering from AD.

## Supplementary Information

**Additional file 1.** ADNM 20 items – Trouble de l’Adaptation Nouveau Module 20.

**Additional file 2.** Visual analogic scales.

## Data Availability

The datasets used and/or analysed during the current study are available from the corresponding author on reasonable request.

## Abbreviation

AD: Adjustment disorder
