# Supplementary material for: Validation of the French ADNM-20 in the assessment of emotional difficulties resulting from COVID-19 quarantine and outbreak
Source: BMC Psychol. 2021 Nov 13;9:180. doi: 10.1186/s40359-021-00683-7 (PMC8590117; doi:10.1186/s40359-021-00683-7)
Supplement: Supplementary file 1 — Additional file 1. ADNM 20 items – Trouble de l’Adaptation Nouveau Module 20. [file 40359_2021_683_MOESM1_ESM.docx]

**Supplementary Material 1**

**ADNM 20 items – Trouble de l’Adaptation Nouveau Module 20**

Voici ci-dessous une liste d’évènements de vie stressants. Merci d’indiquer les évènements qui vous sont arrivés durant les deux dernières années et qui sont actuellement un très lourd fardeau pour vous, ou qui ont été un fardeau pour vous ou cours des 6 derniers mois.

Vous pouvez indiquer plusieurs évènements s’ils s’appliquent à vous.

|  | **Évènement** | **Date (Mois / Année)** |
| --- | --- | --- |
| □ | 1. Divorce / séparation | …… / …… |
| □ | 1. Conflits familiaux | de …… / …… à …… / …… |
| □ | 1. Conflits au travail | de …… / …… à …… / …… |
| □ | 1. Conflits avec le voisinage | de …… / …… à …… / …… |
| □ | 1. Maladie d’un être cher | de …… / …… à …… / …… |
| □ | 1. Décès d’un être cher | …… / …… |
| □ | 1. Adaptation à la retraite | …… / …… |
| □ | 1. Chômage | de …… / …… à …… / …… |
| □ | 1. Trop / pas assez de travail | de …… / …… à …… / …… |
| □ | 1. Pression pour respecter des délais / pression du temps | de …… / …… à …… / …… |
| □ | 1. Emménager dans un nouveau logement | …… / …… |
| □ | 1. Problèmes financiers | de …… / …… à …… / …… |
| □ | 1. Propre maladie grave | de …… / …… à …… / …… |
| □ | 1. Accident grave | …… / …… |
| □ | 1. Agression | …… / …… |
| □ | 1. Fin d’une activité de loisir importante | de …… / …… à …… / …… |
| □ | 1. Confinement suite à une épidémie | de …… / …… à …… / …… |
| □ | 1. Tout autre évènement de vie stressant (merci de préciser) : …………………………………….. | de …… / …… à …… / …… |
| □ | 1. Tout autre évènement de vie stressant (merci de préciser) : …………………………………….. | de …… / …… à …… / …… |

Les évènements que vous venez d’indiquer peuvent avoir de nombreuses conséquences sur notre bien-être et comportement.

Merci d’indiquer ci-dessous quel(s) événement(s) a(ont) été le(s) plus éprouvant(s) :

…………………………………………………………………………………………………

…………………………………………………………………………………………………

…………………………………………………………………………………………………

…………………………………………………………………………………………………

Les évènements que vous venez d’indiquer peuvent avoir de nombreuses conséquences sur notre bien-être et comportement. Ci-dessous vous trouvez différentes affirmations à propos de réactions que ce type d’évènements peut déclencher. Tout d’abord, merci d’indiquer à quelle fréquence les différentes affirmations s’appliquent à vous (de ‘Jamais’ à ‘Souvent’).

Ensuite, merci d’indiquer depuis combien de temps vous avez eu cette réaction. Cela peut être de moins d’un mois (<1 mois), entre un mois et une demi-année (1-6 mois), ou plus de 6 mois (6 mois-2 ans). Cela peut ne pas être très simple à indiquer, mais merci d’essayer de donner une estimation approximative de la durée de la réaction.

Si vous n’avez pas indiqué d’évènement de vie stressant dans la liste précédente, alors vous pouvez ignorer les questions qui suivent.

|  | **Jamais (1)** | **Rarement (2)** | **Parfois (3)** | **Souvent (4)** |
| --- | --- | --- | --- | --- |
| 1. Depuis la situation stressante, je me sens déprimé·e et triste | □ | □ | □ | □ |
|  | <1 mois 1–6 mois 6 mois-2 ans | | | |
| 2. Je dois penser à la situation stressante de manière répétée | □ | □ | □ | □ |
|  | <1 mois 1–6 mois 6 mois-2 ans | | | |
| 3. J’essaie d’éviter de parler de la situation stressante chaque fois que cela est possible | □ | □ | □ | □ |
|  | <1 mois 1–6 mois 6 mois-2 ans | | | |
| 4. Je dois penser à la situation stressante beaucoup et cela est un lourd fardeau pour moi | □ | □ | □ | □ |
|  | <1 mois 1–6 mois 6 mois-2 ans | | | |
| 5. Je fais rarement les activités qui me plaisaient avant | □ | □ | □ | □ |
|  | <1 mois 1–6 mois 6 mois-2 ans | | | |
| 6. Si je pense à la situation stressante, je me retrouve dans un réel état d’anxiété | □ | □ | □ | □ |
|  | <1 mois 1–6 mois 6 mois-2 ans | | | |
| 7. J’évite certaines choses qui peuvent me rappeler la situation stressante | □ | □ | □ | □ |
|  | <1 mois 1–6 mois 6 mois-2 ans | | | |
| 8. Je suis nerveux.se et agité·e depuis la situation stressante | □ | □ | □ | □ |
|  | <1 mois 1–6 mois 6 mois-2 ans | | | |
| 9. Depuis la situation stressante, je me mets en colère bien plus rapidement qu'auparavant, même pour de petites choses | □ | □ | □ | □ |
|  | <1 mois 1–6 mois 6 mois-2 ans | | | |
| 10. Depuis la situation stressante, je trouve difficile de me concentrer sur certaines choses | □ | □ | □ | □ |
|  | <1 mois 1–6 mois 6 mois-2 ans | | | |
| 11. J’essaie de rejeter la situation stressante de ma mémoire | □ | □ | □ | □ |
|  | <1 mois 1–6 mois 6 mois-2 ans | | | |
| 12. J’ai remarqué que je deviens plus irritable à cause de la situation stressante | □ | □ | □ | □ |
|  | <1 mois 1–6 mois 6 mois-2 ans | | | |
| 13. J’ai constamment des souvenirs de la situation stressante et je ne peux rien faire pour les arrêter | □ | □ | □ | □ |
|  | <1 mois 1–6 mois 6 mois-2 ans | | | |
| 14. J’essaie de supprimer mes sentiments car ils sont un fardeau pour moi | □ | □ | □ | □ |
|  | <1 mois 1–6 mois 6 mois-2 ans | | | |
| 15. Mes pensées tournent souvent autour de tout ce qui est relié à la situation stressante | □ | □ | □ | □ |
|  | <1 mois 1–6 mois 6 mois-2 ans | | | |
| 16. Depuis la situation stressante, j’ai peur de faire certaines choses ou de me retrouver dans certaines situations | □ | □ | □ | □ |
|  | <1 mois 1–6 mois 6 mois-2 ans | | | |
| 17. Depuis la situation stressante, je n’aime pas aller au travail ou faire les tâches quotidiennes nécessaires | □ | □ | □ | □ |
|  | <1 mois 1–6 mois 6 mois-2 ans | | | |
| 18. Je me sens découragé·e depuis la situation stressante et j’ai peu d’espoir dans l’avenir. | □ | □ | □ | □ |
|  | <1 mois 1–6 mois 6 mois-2 ans | | | |
| 19. Depuis l’évènement stressant, je ne peux plus dormir correctement | □ | □ | □ | □ |
|  | <1 mois 1–6 mois 6 mois-2 ans | | | |
| 20. Dans l’ensemble, la situation cause une détérioration importante de ma vie sociale et professionnelle, de mon temps de loisirs, et des autres domaines importants de fonctionnement | □ | □ | □ | □ |
|  | <1 mois 1–6 mois 6 mois-2 ans | | | |

| **Symptômes centraux** | | | | | |
| --- | --- | --- | --- | --- | --- |
|  | Préoccupations | 2 | 4 | 13 | 15 |
|  | Échec d’adaptation | 10 | 17 | 19 | 20 |
| **Symptômes accessoires** | | | | | |
|  | Évitement | 3 | 7 | 11 | 14 |
|  | Humeur dépressive | 1 | 5 | 18 |  |
|  | Anxiété | 6 | 16 |  |  |
|  | Trouble de l’impulsion | 8 | 9 | 12 |  |

**References**

- Einsle, F., Köllner, V., Dannemann, S., & Maercker, A. (2010). Development and validation of a self-report for the assessment of adjustment disorders. *Psychology, Health & Medicine*, 15(5), 584-595.
- Glaesmer, H., Romppel, M., Braehler, E., Hinz, A., & Maercker, A. (2015). Adjustment Disorder as proposed for ICD-11: Dimensionality and Symptom Differentiation. *Psychiatry Research*, 229, 940-948.
- Lorenz, L. (2016). Diagnostik von Anpassungsstörungen. Ein Fragebogen zum neuen ICD-11-Modell. Wiesbaden: Springer Fachmedien.
- Lorenz, L., Bachem, R.C., Maercker, A. (2016). The Adjustment Disorder–New Module 20 as a screening instrument: Cluster analysis and cut-off values. *Int J Occup Environ Med*, 7, 215-220.
- Maercker, A., Einsle, F., Köllner, V. (2007). Adjustment disorders as stress response syndromes: A new diagnostic concept and its exploration in a medical sample. *Psychopathology*, 40, 135-146
