# Supplementary material for: Validation of the French ADNM-20 in the assessment of emotional difficulties resulting from COVID-19 quarantine and outbreak
Source: BMC Psychol. 2021 Nov 13;9:180. doi: 10.1186/s40359-021-00683-7 (PMC8590117; doi:10.1186/s40359-021-00683-7)
Supplement: Supplementary file 2 — Additional file 2. Visual analogic scales. [file 40359_2021_683_MOESM2_ESM.docx]

**Supplementary Material 2**

Q1: Are you able to keep yourself busy?

Q2: Are you able to keep a good sleep rhythm?

Q3: Are you able to keep a stable alimentation?

Q4: Are you able to see the positive aspects of quarantine?

Q5: Are you able to relax during quarantine?

Q6: Are you able to manage worries related to the outbreak?

Q7: Are you spending the quarantine with someone (Yes or No)? Did you succeed to communicate with the other?

Q8: Are you able to obtain clear information about outbreak the evolution?

Q9: Are you able to manage the time you spend in front of screens?

Q10: Are you able to keep a regular physical activity?

Q11: Are you able to keep social contact?

Q12: Are you involved in joined activity?

Q13: Do you consume more psychoactive substances than usually?

Q14: Do you keep your professional activity?

Q15: Do you respect quarantine?
